# Impact of Feature Choice on Machine Learning Classification of Fractional Anomalous Diffusion

**DOI:** 10.3390/e22121436

**Published:** 2020-12-19

**Authors:** Hanna Loch-Olszewska, Janusz Szwabiński

**Affiliations:** Faculty of Pure and Applied Mathematics, Hugo Steinhaus Center, Wrocław University of Science and Technology, 50-370 Wrocław, Poland

**Keywords:** anomalous diffusion, machine learning classification, feature engineering

## Abstract

The growing interest in machine learning methods has raised the need for a careful study of their application to the experimental single-particle tracking data. In this paper, we present the differences in the classification of the fractional anomalous diffusion trajectories that arise from the selection of the features used in random forest and gradient boosting algorithms. Comparing two recently used sets of human-engineered attributes with a new one, which was tailor-made for the problem, we show the importance of a thoughtful choice of the features and parameters. We also analyse the influence of alterations of synthetic training data set on the classification results. The trained classifiers are tested on real trajectories of G proteins and their receptors on a plasma membrane.

## 1. Introduction

Starting with the pioneering experiment performed by Perrin [1], the quantitative analysis of microscopy images has become an important technique for various disciplines ranging from physics to biology. Over the last century, it has evolved to what is now known as single-particle tracking (SPT) [2,3,4]. In recent years, SPT has gained popularity in the biophysical community. The method serves as a powerful tool to study the dynamics of a wide range of particles including small fluorophores, single molecules, macromolecular complexes, viruses, organelles and microspheres [5,6]. Processes such as microtubule assembly and disassembly [7], cell migration [8], intracellular transport [9,10] and virus trafficking [11] have been already successfully studied with this technique.

A typical SPT experiment results in a series of coordinates over time (also known as “trajectory”) for every single particle, but it does not provide any directed insight into the dynamics of the investigated process by itself. Mobility patterns of particles encoded in their trajectories have to be extracted in order to relate individual trajectories to the behavior of the system at hand and the associated biological process [12]. The analysis of SPT trajectories usually starts with the detection of a corresponding motion type of a particle, because this information may already provide insights into mechanical properties of the particle’s surrounding [13]. However, this initial task usually constitutes a challenge due to the stochastic nature of the particles’ movement.

There are already several approaches to analyse the mobility patterns of particles. The most commonly used one is based on the mean square displacement (MSD) of particles [10,14,15,16,17]. The idea behind this method is quite simple: a MSD curve (i.e., an average square displacement as a function of the time lag) is quantified from a single experimental trajectory and then fitted with a theoretical expression [18]. A linear best fit indicates normal diffusion (Brownian motion) [19], which corresponds to a particle moving freely in its environment. Such a particle neither interacts with other distant particles nor is hindered by any obstacles. If the fit is sublinear, the particle’s movement is referred to as subdiffusion. It is appriopriate to represent particles moderated by viscoelastic properties of the environment [20], particles which hit upon obstacles [21,22] or trapped particles [9,23]. Finally, a superlinear MSD curve means superdiffusion, which relates to the motion of particles driven by molecular motors. This type of motion is faster than the linear case and usually in a specific direction [24].

Although popular in the SPT community, the MSD approach has several drawbacks. First of all, experimental uncertainties introduce a great amount of noise into the data, making the fitting of mathematical models challenging [10,14,25,26]. Moreover, the observed trajectories are often short, limiting the MSD curves to just a few first time lags. In this case, distinguishing between different theoretical models may not be feasible. To overcome these problems, several analytical methods that improve or go beyond MSD have already been proposed. The optimal least-square fit method [10], the trajectory spread in space measured with the radius of gyration [27], the van Hove displacements distributions [28], self-similarity of trajectory using different powers of the displacement [29] or the time-dependent directional persistence of trajectories [30] are examples of methods belonging to the first category. They may be combined with the results of the pure MSD analysis to improve the outcome of classification. The distribution of directional changes [31], the mean maximum excursion method [32] and the fractionally integrated moving average (FIMA) framework [33] belong to the other class. They allow efficient replacement of the MSD estimator for classification purposes. Hidden Markov models (HMM) turned out to be quite useful in heterogeneity checking within single trajectories [34,35] and in the detection of confinement [36]. Classification based on hypothesis testing, both relying on MSD and going beyond this statistics, has been shown to be quite successful as well [26,37].

In the last few years, machine learning (ML) has started to be employed for the analysis of single-particle tracking data. In contrast to standard algorithms, where the user is required to explicitly define the rules of data processing, ML algorithms can learn those rules directly from series of data. Thus, the principle of ML-based classification of trajectories is simple: an algorithm learns by adjusting its behavior to a set of input data (trajectories) and corresponding desired outputs (real motion types, called the ground truth). These input–output pairs constitute the training set. A classifier is nothing but a mapping between the inputs and the outputs. Once trained, it may be used to predict the motion type of a previously unseen sample.

The main factor limiting the deployment of ML to trajectory analysis is the availability of high-quality training data. Since the data collected in the experiments is not really provable (otherwise, we would not need any new classification method), synthetic sets generated with computer simulations of different diffusion models are usually used for training.

Despite the data-related limitations, several attempts at ML-based analysis of SPT experiments have been already carried out. The applicability of the Bayesian approach [18,38,39], random forests [40,41,42,43], neural networks [44] and deep neural networks [41,45,46] was extensively studied. The ultimate goal of those works was the determination of the diffusion modes. However, some of them went beyond the pure classification and focused on extraction of quantitative information about the trajectories (e.g., the anomalous exponent [42,45]).

In one of our previous papers, we compared two different ML approaches to classification [41]. Feature-based methods do not use raw trajectories as input for the classifiers. Instead, they require a set of human-engineered features, which are then used to feed the algorithms. In contrast, deep learning (DL) methods extract features directly from raw data without any effort from human experts. In this case, the representation of data is constructed automatically and there is no need for complex data preprocessing. Deep learning is currently treated as the state-of-the-art technology for automatic data classification and slightly overshadows the feature-based methods. However, from our results, it follows that the latter are still worth to consider. Compared to DL, they may arrive at similar accuracies in much shorter training times, are usually easier to interpret, allow to work with trajectories of different lengths in a natural way and often do not require any normalisation of data. The only drawback of those methods is that there is not a universal set of features that works well for trajectories of any type. Choosing the features is challenging and may have an impact on the classification results.

In this paper, we would like to elaborate on the choice of proper features to represent trajectories. Comparing classifiers trained on the same set of trajectories, but with slightly different features, we will address some of the challenges of feature-based classification.

The paper is structured as follows. In Section 2, we briefly introduce the concept of anomalous diffusion and present the stochastic models that we chose to model it. In Section 3, methods and data sets used in this work are discussed. The results of classification are extensively analysed in Section 4. In the last section, we summarise our findings.

## 2. Anomalous Diffusion and Its Stochastic Models

Non-Brownian movements that exhibit non-linear mean squared displacement can be described by multiple models, depending on some specific properties of the corresponding trajectories. The most popular models are the continuous-time random walk (CTRW) [9], random walks on percolating clusters (RWPC) [47,48], fractional Brownian motion (FBM) [49,50,51], fractional Lévy α-stable motion (FLSM) [52], fractional Langevin equation (FLE) [53] and autoregressive fractionally integrated moving average (ARFIMA) [54].

In this paper, we follow the model choice described in [26,37,43]—namely, we use FBM, the directed Brownian motion (DBM) [55] and Ornstein–Uhlenbeck (OU) processes [56]. With the particular choice of the parameters, all these models simplify to the classical Brownian motion (i.e., normal diffusion).

The FBM is the solution of the stochastic differential equation

(1) $$dX_{t}^{i}=\sigma dB_{t}^{H,i},\;\;i=1,2,$$

where σ>0 is the scale coefficient, which relates to the diffusion coefficient *D* via $\sigma =\sqrt{2D}$, H∈(0,1) is the Hurst parameter and $B_{t}^{H}$ is a continuous-time, zero-mean Gaussian process starting at zero, with the following covariance function

(2) $$E\left(B_{t}^{H}B_{s}^{H}\right)=\frac{1}{2}\left({\left|t\right|}^{2H}+{\left|s\right|}^{2H}-{\left|t-s\right|}^{2H}\right).$$

The value of *H* determines the type of diffusion in the process. For $H<\frac{1}{2}$, FBM produces subdiffusion. It corresponds to a movement of a particle hindered by mobile or immobile obstacles [57]. For $H>\frac{1}{2}$, FBM generates superdiffusive motion. It reduces to the free diffusion at $H=\frac{1}{2}$.

The directed Brownian motion, also known as the diffusion with drift, is the solution to

(3) $$dX_{t}^{i}=v_{i}dt+\sigma dB_{t}^{1/2,i},\;\;i=1,2,$$

where $v=\left(v_{1},v_{2}\right)\in R^{2}$ is the drift parameter and σ is again the scale parameter. For v=0, it reduces to normal diffusion. For other choices of *v*, it generates superdiffusion related to an active transport of particles driven by molecular motors.

The Ornstein–Uhlenbeck process is often used as a model of a confined diffusion (a subclass of subdiffusion). It describes the movement of a particle inside a potential well and can be determined as the solution to the following stochastic differential equation:

(4) $$dX_{t}^{i}=-\lambda_{i}\left(X_{t}^{i}-\theta_{i}\right)dt+\sigma dB_{t}^{1/2,i},\;\;i=1,2,\;\;\theta_{i}\in R.$$

The parameter $\theta =(\theta_{1},\theta_{2})$ is the long-term mean of the process (i.e., the equilibrium position of a particle), $\lambda =(\lambda_{1},\lambda_{2})$ is the value of a mean-reverting speed and and σ is again the scale parameter. If there is no mean reversion effect, i.e., $\lambda_{i}=0$, OU reduces to normal diffusion.

## 3. Methods and Used Data Sets

In this paper, we discuss two feature-based classifiers: random forest (RF) and gradient boosting (GB) [58]. The term feature-based relates to the fact that the corresponding algorithms do not operate on raw trajectories of a process. Instead, for each trajectory a vector of human-engineered features is calculated and then used as input for the classifier. This approach for the diffusion mode classification has already been used in [41,42,43,45], but here, we propose a new set of features, which gives better results on synthetic data sets.

Both RF and GB are examples of ensemble methods, which combine multiple classifiers to obtain better predictive performance. They use decision trees [59] as base classifiers. A single decision tree is fairly simple to build. The original data set is split into smaller subsets based on values of a given feature. The process is recursively repeated until the resulting subsets are homogeneous (all samples from the same class) or further splitting does not improve the classification performance. A splitting feature for each step is chosen according to Gini impurity or information gain measures [58].

A single decision tree is popular among ML methods due to the ease of its interpretation. However, it has several drawbacks that disqualify it as a reliable classifier: it is sensitive to even small variations of data and prone to overfitting. Ensemble methods combining many decision trees help to overcome those drawbacks while maintaining most of the advantages of the trees. A multitude of independent decision trees is constructed by making use of the bagging idea with the random subspace method [60,61,62] to form a random forest. Their prediction is aggregated and the mode of the classes of the individual trees is taken as the final output. In contrast, the trees in gradient boosting are built in a stage-wise fashion. At every step, a new tree learns from mistakes committed by the ensemble. GB is usually expected to perform better than RF, but the latter one may be a better choice in case of noisy data.

In this work, we used implementations of RF and GB provided by the scikit-learn Python library [63]. The performance of the classifiers was evaluated with the common measures including accuracy, precision, recall, F1 score and confusion matrices (although the information given by those measures is to some extent redundant, we decided to use all of them due to their popularity). The accuracy is a percentage of correct predictions among all predictions, that is a general information about the performance of a classifier (reliable in case of the balanced data set). The precision and recall give us a bit more detailed information for each class. The precision is a ratio of the correct predictions to all predictions in that class (including the cases falsely assigned to this class). On the other hand, the recall (also called sensitivity or true positive rate) is the ratio of correct predictions of that class to all members of that class (including the ones that were falsely assigned to another class). The F1 score is a harmonic mean of precision and recall, resulting in high value only if both precision and recall are high. Finally, the confusion matrices show detailed results of classification: element $c_{i,j}$ of matrix C is the percentage of the observations from class *i* assigned to class *j* (a row presents actual class, while the column presents predicted class).

The Python codes for the data simulation, features calculation, models preparation and performance calculation are available at Zenodo (see Supplementary Materials).

### 3.1. Features Used for Classification

As already mentioned above, both ensemble methods require vectors of human-engineered features representing the trajectories as input. In some sense, those methods may be treated as a kind of extension to the statistical methods usually used for classification purposes. Instead of conducting a statistical testing procedure of diffusion based on one statistic, what is often the case, we can combine several statistics with each other bu turning them into features, which are then used to train a classifier. This could be of particular importance in situations, when single statistics yield results differing from each other (cf. [43]). It should be mentioned, however, that choosing the right features is a challenging task. For instance, we have already shown in [41] that classifiers trained with a popular set of features do not generalise well beyond the situations encoutered in the training set. Thus, great attention needs to be paid to the choice of the input features to machine learning classifiers as well. They ought to cover all the important characteristics of the process, but at the same time, they should contain the minimal amount of unnecessary information, as each redundant piece of data causes noise in the classification or may lead to overfitting, for example (for a general discussion concerning a choice of features, see, for instance, [64]).

Based on the results in [41,43], we decided to use the following features in our analysis, hereinafter referred to as Set A:

- Anomalous exponent α, fitted to the time-averaged mean square displacement (TAMSD). This exponent relates to the Hurst parameter in Equation (1) via α=2H.
- Diffusion coefficient *D*, fitted to TAMSD.
- Mean squared displacement ratio, characterising the shape of a MSD curve. In general, it is given with the formula
  $$\kappa \left(n_{1},n_{2}\right)=\frac{\frac{1}{N-n_{1}}\sum_{i=1}^{N-n_{1}}{\left|X_{i+n_{1}}-X_{i}\right|}^{2}}{\frac{1}{N-n_{2}}\sum_{i=1}^{N-n_{2}}{\left|X_{i+n_{2}}-X_{i}\right|}^{2}}-\frac{n_{1}}{n_{2}},$$
  where $n_{1}<n_{2}$. In this work, we set $n_{2}=n_{1}+1$ and averaged the output over $n_{1}$. In other words, we used ($n_{1}$ replaced by *n* for convenience):
  (5) $$\kappa =\frac{1}{N-1}\sum_{n=1}^{N-1}\kappa \left(n,n+1\right).$$
- Efficiency, calculated as
  (6) $$E=\frac{\left|X_{N-1}-X_{0}\right|}{\left(N-1\right)\sum_{i=1}^{N-1}{\left|X_{i}-X_{i-1}\right|}^{2}},$$
  which measures the linearity of a trajectory.
- Straightness, a measure of the average direction change between subsequent steps, calculated as:
  (7) $$S=\frac{\left|X_{N-1}-X_{0}\right|}{\left(N-1\right)\sum_{i=1}^{N-1}\left|X_{i}-X_{i-1}\right|}.$$
- The value of empirical velocity autocorrelation function [65] of lag 1 in point n=1, that is
  $$\chi =\frac{1}{N-2}\sum_{i=1}^{N-2}\left(X_{i+2}-X_{i+1}\right)\cdot \left(X_{i+1}-X_{i}\right).$$
- Maximal excursion, given by the formula
  (8) $$ME=\frac{\max(X_{i+1}-X_{i})}{X_{N-1}-X_{0}}.$$
  It is inspired by the mean maximal excursion (MME) [32], detecting the jumps that are long as compared to the overall displacement.
- The statistics based on *p*-variation [52]:
  $$V_{m}^{\left(p\right)}=\sum_{i=0}^{N/m-1}{\left|X_{(i+1)m}-X_{im}\right|}^{p}.$$
  The usefulness of this statistic to recognition of the fractional Lévy stable motion (including fractional Brownian motion) was shown in [52]. We introduce a quantity that verifies if for any *p* the function $V_{m}^{\left(p\right)}$ of the variable *m* changes the monotonicity. We provide the information if for the highest value of *p* such that $V_{m}^{\left(p\right)}$ does change the monotonicity, it is convex or concave. In short, we analyse $V_{m}^{\left(p\right)}$ as a function of *m* to provide one the following values:
  (9) $$P=\left\{\begin{array}{cc} 0 & \mathrm{if}\mathrm{it}\mathrm{does}\mathrm{not}\mathrm{change}\mathrm{the}\mathrm{monotonicity},\\ 1 & \mathrm{if}\mathrm{it}\mathrm{is}\mathrm{convex}\mathrm{for}\mathrm{the}\mathrm{highest}p\mathrm{for}\mathrm{which}\mathrm{it}\mathrm{is}\mathrm{not}\mathrm{monononuous},\\ -1 & \mathrm{if}\mathrm{it}\mathrm{is}\mathrm{concave}\mathrm{for}\mathrm{the}\mathrm{highest}p\mathrm{for}\mathrm{which}\mathrm{it}\mathrm{is}\mathrm{not}\mathrm{monononuous}. \end{array}\right.$$

The first five features were already used in [41]. It should also be mentioned here that three of them are based on MSD curves. There is one important point to consider while calculating the curves, namely the maximum time lag. If not specified otherwise, we will use the lag equal to 10% of each trajectory’s length. Since this choice is not obvious and may impact the classification performance, we will discuss the sensitivity of classifiers’ accuracies to different choices of the lag in Section 4.5.

Apart from the set of features presented above, denoted Set A, we are going to analyse two other sets: the one used in [40,41], referred as Set B, and the one proposed in [43] (set C). The lists of features used in each set are given in Table 1 (for their exact definition, please see the mentioned references). Sets A and B have several features in common. The link between sets A and C is not so apparent, but the maximal excursion and *p*-variation-based statistics play in the description of trajectories a role similar to the standardised maximum distance and the exponent of power function fitted to *p*-variation, respectively.

Following [41], we consider four classifiers for each set of features: RF and GB classifiers built with the full set (labelled as “with *D*”) and with a reduced one after the removal of the diffusion constant *D* (“no *D*”).

### 3.2. Synthetic Data

Unlike the explicitly programmed methods, machine learning algorithms are not ready-made solutions for arbitrary data. Instead, an algorithm needs to be firstly fed with a reasonable amount of data (so-called training data) that should contain the main characteristics of the process under investigation in order to find and learn some hidden patterns. As the classifier is not able to extract any additional patterns from previously unseen samples after this stage, its performance is highly dependent on the quality of the training data. Hence, the training set needs to be complete in some sense.

First, we created our main data set, which will be referred to as the base data set for the remainder of this paper. It is analogous to the one used in [43]. We generated a number of 2D trajectories according to the three diffusion models described in Section 2, with no correlations between the coordinates. A single trajectory can be denoted as

(10) $$X_{n}=\left(X_{t_{0}},X_{t_{1}},\cdots ,X_{t_{N}}\right),$$

where $X_{t_{i}}=\left(X_{t_{i}}^{1},X_{t_{i}}^{2}\right)\in R^{2}$ is the position of the particle at time $t_{i}=t_{0}+i\Delta t$, i=0,1,⋯,N. We kept the lag Δt between two consecutive observations constant.

The details of our simulations are summarised in Table 2. In total, 120,000 trajectories have been produced, 40,000 for each diffusion mode, in order to balance the data set. The length of the trajectories was randomly chosen from the range between 50 and 500 steps to mimic typical observations in experiments. We set $\sigma =1\;\mu m\;s^{-1/2}$ and Δt=1s.

Since the normal diffusion can be generated by a particular choice of the models’ parameters (H=0.5 for FBM, v=0 for DBM and λ=0 for OU), it is almost indistinguishable from the anomalous diffusion generated with the parameters in the vicinity of those special values. The addition of the noise complicates the problem even more. Thus, following [43], we introduced a parameter *c* that defines a range in which a weak sub- or superdiffusion should be treated as a normal one. Although introduced here at a different level, it bears resemblance to the cutoff *c* used in [37].

Apart from the base data set, we are going to use several auxiliary ones to elaborate on different aspects of the feature choice. In Section 4.3, we will work with a training set, in which the trajectories from the base one are disturbed with a Gaussian noise to resemble experimental uncertainties. In Section 4.4, we will analyse the performance of classifiers trained on synthetic data generated with σ=0.38, corresponding to the diffusion coefficient $D=0.0715\;\mu m^{2}\;s^{-1}$, which is adequate for the analysis of real data samples. To study the sensitivity of the classifiers to the value of the cutoff *c* in Section 4.6, we will use three further sets with c=0, c=0.001 and c=0.01. In Section 4.7, a synthetic set with σ=2D, where *D* is drawn from the uniform distribution on [1,9] will be used to check how the classifiers cope with the trajectories characterised by heterogeneous mobilities.

For all data sets, the training and testing subset were randomly selected with a 70%/30% ratio.

### 3.3. Empirical Data

**Figure 1:**
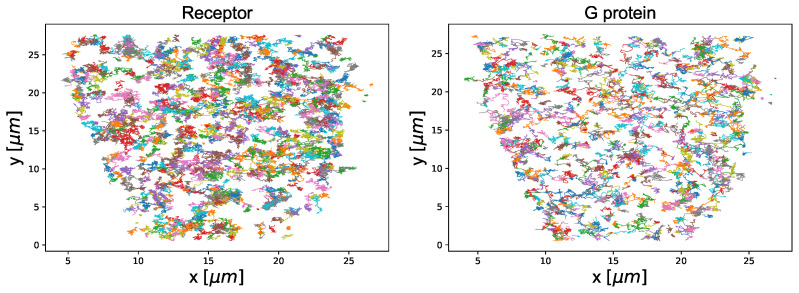
Trajectories of the receptors (**left**) and G proteins (**right**) used as input for the classifiers. Different colors are introduced to indicate different trajectories. The set of the receptors contains 1218 trajectories and the one of G proteins—1037 trajectories. The lengths of the trajectories are from range [50,401], the time step is equal to 28.4 ms and recorded positions are given in μm.

To check how our classifiers work on unseen data, we will apply them to some real data. We decided to use the trajectories of G proteins and G-protein-coupled receptors already analysed in [37,43,66]. To avoid some issues related with short time series, we limited ourselves to trajectories with at least 50 steps only, obtaining 1037 G proteins’ and 1218 receptors’ trajectories. They are visualised in Figure 1.

## 4. Results

The main goal of our work is a comparative analysis of classifiers trained using different sets of features (see Table 1 for their definition). The classifiers were trained and tested on our base data set and the auxiliary data sets, for comparison.

In order to optimise both classification algorithms, we looked for their hyperparameters using the RandomisedSearchCV method from scikit-learn library. It performs a search over values of hyperparameters generated from their distributions (in our case, discrete uniform ones). The term hyperparameter in this context means a parameter required for the construction of the classifier, which has to be set by a human expert before the learning process starts. In general, it influences the performance of the classifier, hence its choice is essential.

### 4.1. Classification Results on Base Data Set Using Proposed Set of Features

We start with the classifiers trained on the base set (see Table 2 for details). We trained four different classifiers: RF and GB for both the full set of attributes (“with D”) and a reduced one (“no D”). Set A of features was used for representation of trajectories. The performance of these classifiers will be treated as a benchmark in our further analysis.

The hyperparameters of the classifiers are presented in Table 3 (for the detailed explanation of each of these parameters, please see [43,58]). It is worth noticing a difference in the ensemble sizes between the full set and the reduced one—in case of the gradient boosting, we observe a ninefold reduction of the number of trees. However, this difference does not reflect in the performance of the classifiers. Taking the number of features into account, the value of the max_depth hyperparameter for RF with *D* is surprisingly high. It seems to be an artifact of the hyperparameter tuning procedure via random grid search. From our analysis (not included in this paper), it follows that this value can be set to 20 without a negative impact on accuracy. Nevertheless, we decided to keep the original result of the automatic hyperparameter tuning in order to treat all of the classifiers on the same footing. We should probably add that the largest tree in RF was 38 levels deep, despite such a high value of the maximum depth.

We begin the analysis of the classifiers by inspecting their accuracies. The results are shown in Table 4. As we can see, both classifiers perform excellently, with more than 95% of correct predictions for the test set. In the case of the training data, GB performs better than RF. However, RF is slightly more accurate on the test set, indicating a small tendency of GB to overfit.

To explain the relatively small differences in the performance between the “with D” and “no D” versions of the classifiers, we may want to look at the importances of features. There are several ways to calculate those importances. We used a method which defines the importance as the decrease in accuracy after a random shuffling of values of one of the features. Results are given in Table 5. Just to recall, features with high importances are the drivers of the outcome. The last important ones might often be omitted, making the classification model faster to fit and predict. The results of the node impurity importances (the total decrease in node impurity caused by a given feature, averaged over all trees in the ensemble [67]) are similar.

It turns out that *D* is the least important feature for RF classifier trained on the full set and the third one with the smallest importance for GB classifier. That is why its removal has a small impact on the accuracy of prediction and why the classifiers trained on the reduced set of features with no *D* are worth considering—we expect them to work better on unseen data having diffusion coefficients different from the one used in the base set. Indeed, its removal does not change the performance of the classifier on the test set (see Table 4). Later in Section 4.7, we will show that in case of the training set with varying *D*, the situation is different: *D* will become more important and excluding it from the set will reduce the accuracy.

The most informative feature in all cases is the velocity autocorrelation function for lag δ=1 at point n=1. It is worth mentioning that this quantity has been already successfully used for the distinction of subdiffusion models [68], but not in the ML context. The anomalous exponent α, which is a standard method for the diffusion mode classification, is the second most important feature for all models, with a significant influence on the results. Thus, it seems that the classifiers distinguish between the models first and then assess the mode of diffusion.

**Figure 2:**
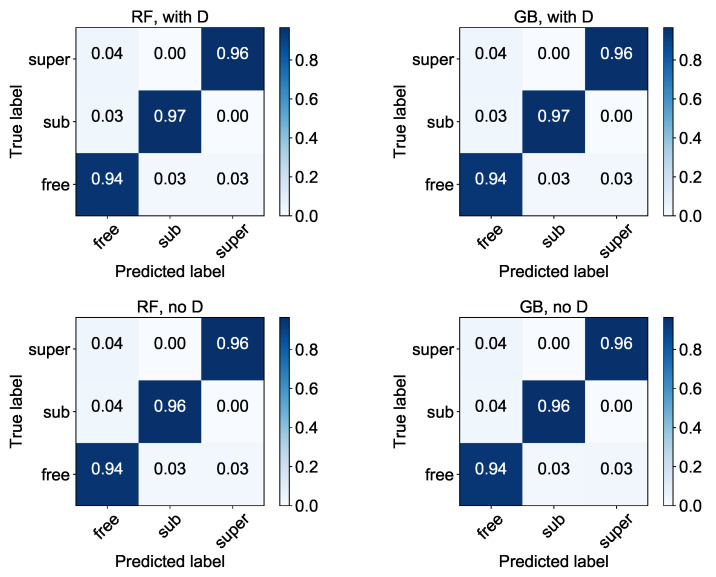
Normalised confusion matrices for classifiers built on base training data (see Table 2) with Set A of features. The “with *D*” (top row) and “no *D*” (bottom row) labels refer to the full and reduced (after removal of *D*) sets of features, respectively. All results are rounded to two decimal digits.

To get more insight into the detailed performance of the classifiers, their normalised confusion matrices are shown in Figure 2. Please note that the percentages may not sum to 1.0 due to rounding. We see that all models have the biggest problems with the classification of normal diffusion. This is simply due to the fact that the differences between normal diffusion and realizations of weak sub- or superdiffusion are negligible and it is challenging to classify it properly even after introduction of the parameter *c* (the role of which will be studied in more detail in Section 4.6).

The values presented in Figure 2 may be used to calculate the other popular measures of performance: precision, recall and F1 score (see Section 3). The results, rounded to three decimal digits, are summarised in Table 6. Again, we see that the measures point to the highest error rate for the normal diffusion: for the random forest model with *D* as one of the features, only 92.9% of the trajectories classified as normal diffusion were in fact in this class (precision), whereas 94.4% of freely diffusing trajectories were correctly classified (recall). Such a high error rate is related to the mentioned lack of distinctions between the nodes—the normal diffusion is some kind of buffer between subdiffusion and superdiffusion, thus it can be incorrectly classified as one of these two.

### 4.2. Comparison with Other Sets of Features

Below, we show the comparison of the classification results with all considered classifiers (based on three different set of features) on our base data set (Table 2).

In Table 7, the accuracies on the test set are shown, calculated using the tenfold cross-validation method [58]. As the calculation of the accuracy of the classifier is based on the single train/test split, in an unfortunate case, the test set can contain the data with characteristics that have not been seen by classifier during training, and thus the accuracy would be falsely low. The *k*-fold cross-validation is a technique that helps to reduce that bias. The data is randomly split into *k* folds (without replacement) and the model is trained and tested *k* times—each time one fold is the test set, whereas the remaining ones create the training set. The overall accuracy is the mean of the accuracies of each run. The hyperparameters of the particular models are summarised in Table 8 and they were established using the RandomisedSearchCV method again.

In the comparison of all these classifiers, the ones based on the set of features proposed in this article provide the best results on our base synthetic data set. Actually, the choice of features was inspired by two of our previous articles [41,43]. The new set combines the attributes used in those papers: it contains the anomalous exponent α, diffusion coefficient *D*, efficiency, straightness and mean squared displacement ratio that have been used in [41], and the normalised maximal excursion and *p*-variation-based features used in [43].

Nevertheless, we need to underline here that it does not mean that this set of features is the solution for all the classification problems—it simply seems to be the best choice for such synthetic data set. The lack of universality of feature-based methods was already presented in [41]: the classifiers did not generalise well to samples generated with slightly altered models.

To compare the performance of these models in more details, the values of recall, precision and F1 score are given in Table 9. For the sake of clarity, we only compare the random forest classifiers built on the complete features’ sets (with the diffusion coefficient *D*). For the remaining cases, the behaviour is alike, except for the fact that all measures for classifiers with features as in Set B but without diffusion coefficient *D* are significantly lower than for other classifiers. We would like to underline here that the set of features proposed in Section 3.1 provides the best results in all measures used here. For all classifiers, the results for superdiffusion and subdiffusion are better than for normal diffusion class, what is understandable, as the only kind of error that occurs is the misclassification of anomalous diffusion trajectories as the normal diffusion. In case of normal diffusion, a part of misclassified trajectories is labelled as superdiffusion, and another part is labelled as subdiffusion.

### 4.3. Adding Noise

The results on our base data set are promising, but, unfortunately, real data are more challenging to classify, as they usually contain some noise and/or measurement error. Thus, we added a random Gaussian noise with zero mean and standard deviation $\sigma_{Gn}$ to our trajectories. In order to control the noise amplitude with respect to standard deviation of a process, we followed the idea used in [40,41,43], namely setting a random signal-to-noise ratio instead of $\sigma_{Gn}$. The signal-to-noise ratio is defined as

(11) $$Q=\left\{\begin{array}{cc} \frac{\sqrt{D\Delta t+v^{2}\Delta t^{2}}}{\sigma_{Gn}} & \mathrm{for}\;\mathrm{DBM},\\ \frac{\sqrt{D\Delta t}}{\sigma_{Gn}} & \mathrm{otherwise}, \end{array}\right.$$

where $v=\sqrt{v_{1}^{2}+v_{2}^{2}}$. The value of $\sigma_{Gn}$ was calculated for each trajectory separately, based on the random value of *Q* drawn from the uniform distribution on interval $\left[1,9\right]$.

The accuracies of the classifiers trained on the data set with noise are given in Table 10. It is worth comparing the results with Table 4—there is a decrease of the accuracy, especially in case of the reduced set of features (“no *D*”), but both methods still classify the diffusion modes well. Nevertheless, in this case, it turns out that the inclusion of the diffusion coefficient *D* as one of the features is important. Still, for our synthetic data set with noise, the features in Set A seem to describe the characteristics of the used processes most precisely.

### 4.4. Empirical Data

In order to present the methods in a practical context, we are going to apply the classifiers from Section 4.1 and Section 4.3 to real G protein data (see Section 3.3). Additionaly, to follow the approach from [43], we will consider additional classifiers fed with the data set similar to the base one, but with σ=0.38, since this value corresponds to the mean diffusion coefficient of the real data sample ($D=0.0715\;\mu m^{2}s^{-1}$). Accuracies of the additional classifiers are shown in Table 11. Interestingly, they are slightly better than the ones for the base set. It seems that the change of the scale parameter positively influenced the ranges of other characteristics, resulting in an increased accuracy (it worked as implicit feature engineering in the absence of data normalization).

Before we start to analyse the results for real data, there are several points to consider. First, it should be emphasised once again that the data collected in experiments is not provable. Since the ground truth is missing, we cannot really choose the best among the classifiers. We just could use some additional information about the G proteins in order to indicate if the classifiers work reasonably or not. Second, real trajectories are often heterogeneous, meaning that a particle may change its type of motion within a single trajectory [69]. Thus the classifiers fed with homogeneous synthetic data may be not the best choice to work with such data.

In Table 12, Table 13 and Table 14, we show the results of classification of real data with the base classifiers, the ones with the noise and the ones with σ=0.38, respectively. In all three cases, we considered only the “with *D*” classifiers (for the justification, see Section 4.7). The results obtained with the classifiers trained on different data sets vary slightly, but they agree on a small percentage of superdiffusive trajectories. This is somehow expected from the biological background: during their movement, the G proteins and G-protein-coupled receptors pair, spending some amount of time immobilised. In the same time, there is no evidence of any other force that can accelerate the movement.

On our base data set, the classifiers based on Set A label most of both G proteins’ and G protein-coupled receptors’ trajectories as subdiffusion (64–84%, depending on particle type and method). This is somewhat in between the results of classifiers based on Set B and Set C, where the former point to subdiffusion more frequently, while the latter apply only in 52–59% of cases.

Comparing the behaviour of the classifiers based on the different data sets used for training, we can see that the classifiers built on the Set C are the most stable in some sense—they yield similar results independently of the training data, indicating to a significant fraction of subdiffusive and freely diffusing trajectories. For the new proposed set of features, Set A, as well as for Set B, the introduction of noise does not alter the classification significantly, but the decrease of the scale of the trajectories in data set (setting σ=0.38) leads to recognition of more trajectories as the normal diffusion, similarly to the *p*-variation-based statistical test proposed in [37]. Alternately, the GB classifier based on Set B and scaled data set classifies a significant percentage of trajectories as superdiffusive, which is rather unexpected.

For the full picture, in Table 15, we also include the results for the classifiers built with the reduced Set A—that is, without diffusion coefficient *D* (“no *D*”). Following the results for the synthetic trajectories, where on the noisy data set the accuracy for the classifiers based on the reduced set of features is smaller (see Table 10), we acknowledge that the results on that data set can be biased. Indeed, such classifiers claim that most of the trajectories exhibit the normal diffusion, whereas the classifiers built on the base and the scaled data set classify them as subdiffusion.

To sum up, all the classifiers identify most trajectories as normal or subdiffusive, but the fraction of both diffusion modes varies between classifiers. The scaling of trajectories in the training data set has introduced significant changes in the results (please compare Table 12 and Table 14), thus the properties of particular features should be further examined (for example, their normalisation). Moreover, in [69], the authors showed that the trajectories in the analysed data set change their character during the time evolution. Different features used in the classifiers probably capture slightly different characteristics of the trajectories; thus, the sensitivity of features for the heterogeneity of movement should be verified.

### 4.5. Influence of MSD Calculation Methods

Some of the features used in our set—that is, the diffusion coefficient *D*, the anomalous exponent α and the mean displacement ratio κ, are based on the time-averaged MSD. This quantity can be highly biased for large lags, as then only a few displacements are included in the calculation of the mean value. Alternately, if we choose to fit the diffusion coefficient or the anomalous exponent to only a few data points (to MSD calculated for a few lags only), the estimation could be biased. This is a known problem in the analysis of the biological data and has already been discussed in [26,70,71].

We have considered the influence of the number of lags on the accuracy of the classifiers and trained them on the base data set with the values of features calculated using 50% or 10% of available TAMSD length. In Table 16, the comparison of these accuracies on the test set is shown, using all three sets of features. For each set, only the “with *D*” variant has been considered. The better results are obtained with the shorter TAMSD curve, but the differences are only slight. Thus, we have set the 10% as the fixed value for all our considerations.

### 4.6. Sensitivity of the Model to Parameter *C*

Up to this point, we used set of synthetic data generated with c=0.1 (see Table 2 for the meaning of *c*). This parameter was used to define ranges, outside of which weak sub- or superdiffusion should be distinguished from the normal one. It is time to analyse the impact of *c* on the prediction performance of our classification models.

In Table 17, the accuracies on the test set of the particular classifiers are presented. The highest value of this metrics for c=0.1 could suggest that it is is the best choice, but there is the other side of a coin—the highest *c* means that more trajectories in the data set were falsely labelled as normal diffusion on the data set simulation stage, despite the fact that they were generated from models with the parameters corresponding to the anomalous diffusion. In Table 18, the values of precision, recall and F1 are shown for the random forest classifier (“with *D*”) trained on each of the analysed sets. Although the precision for the normal diffusion grows with the increasing value of *c*, there is a drop in the recall value between c=0.01 and c=0.1. Inversely, for both modes of anomalous diffusion, the precision drops when changing from c=0.01 and c=0.1. It means that we not only make a base mistake in labelling, falsely labelling some normal trajectories as anomalous ones at the data set generation stage (what is not visible here), but also setting too high value of *c* parameter adds some confusion.

**Figure 3:**
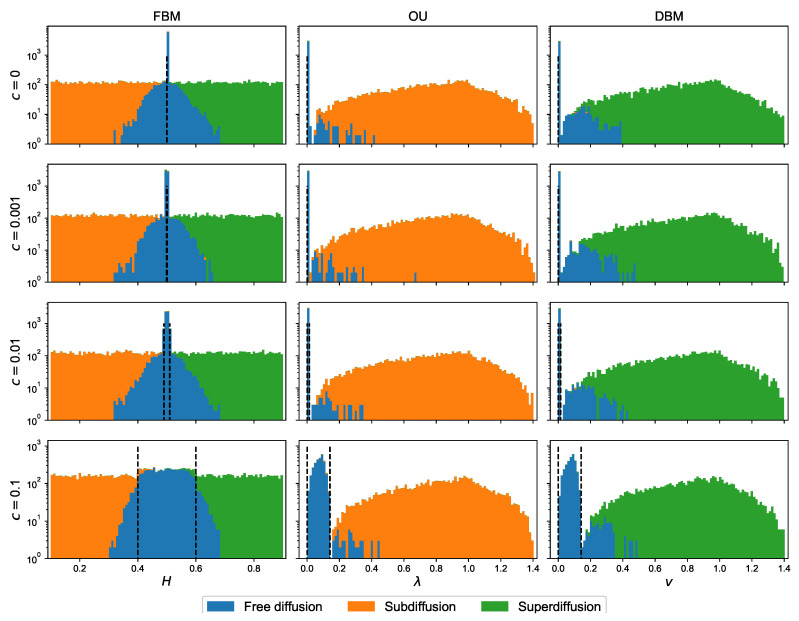
The histograms of assigned labels for different diffusion models, as predicted for the test sets by classifiers built on data sets with different values of parameter *c* with Set A of features. Please mind the logarithmic scale on *y*-axis. The dashed lines bounds the regions for which the normal diffusion was assigned as ground truth despite the real character of trajectories.

The issue is visualised in Figure 3, where the histograms of predicted labels are shown (please mind the logarithmic scale on *y*-axis). The ranges defined by the parameter *C* are indicated with black dashed lines. All observations between the dashed lines were treated as normal diffusion by the classifiers (such label was assigned at the data set generation stage as ground truth). Although for c=0.1 and all diffusion models, the major part of trajectories was classified correctly, the distribution of the normal diffusion label assigned is wider than, for example, c=0.01, especially in the case of fractional Brownian motion. Thus, to diminish the error (understood as an incorrect label in comparison to real diffusion mode, not assigned ground truth label), a smaller value of *c* should be taken—for example, the mentioned c=0.01.

### 4.7. Role of Diffusion Coefficient *D*

Finally, we move to the case in which parameter σ varies between trajectories. The data set for the classification was prepared according to Table 2, but each trajectory was characterised by a random σ value equal to $\sqrt{2D}$, where *D* was drawn from the uniform distribution on the interval $\left[1,9\right]$. The same set of features was used and an additional regularisation was performed in the classifier training procedure.

The accuracy results for such classifiers are shown in Table 19. As one can see, the classifiers are still correct in more than 90% of cases and we can still consider them as useful. Interestingly, the changes in *D* have bigger influence to values than adding noise, introduced in Section 4.3. Thus, our classifiers work better in case of homogeneous environment with a constant diffusion coefficient, and as could be somehow expected, the difference between the classifiers with the diffusion coefficient *D* as a feature and the ones without it is visible, in favour of the all features’ set. Thus, there is no reason to consider the reduced set of features in future research.

**Figure 4:**
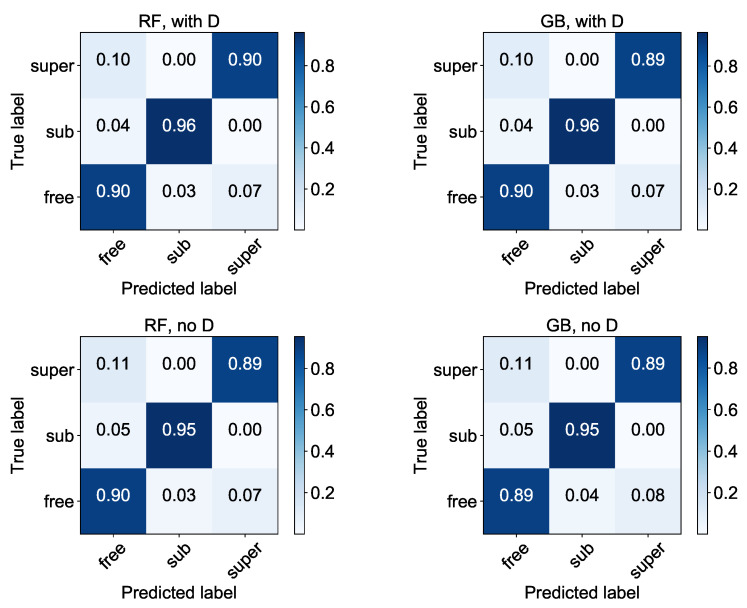
Normalised confusion matrices for classifiers built on training data with varying *D* and Set A of features. All results are rounded to two decimal digits.

In Figure 4, the confusion matrices of the analysed classifiers are shown. There is definitely more confusion between superdiffusion and free diffusion, in both directions, but still there is no misclassification between super- and subdiffusion (what would point to more serious problems with the classification). We think that these results can be even improved with the revision of the diffusion coefficient estimation method.

### 4.8. Beyond Multi-Class Classification

Up to this point, the classifiers were set to output only one among three available classes. However, both RF and GB classifiers are ensemble methods that determine the final output through voting of their base learners (decision trees). That voting can be exploited to provide probabilities of being assigned to each class. Their analysis can help in understanding the classifiers’ behaviour and sources of misclassifications.

**Figure 5:**
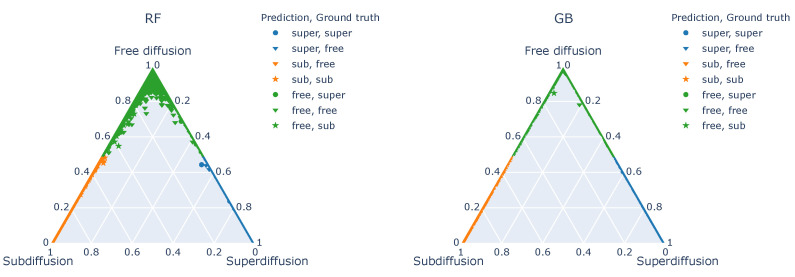
Ternary plots of the class probabilities assigned to the testing data by the classifiers trained on the base data set with Set A of features.

In Figure 5, ternary plots for both random forest and gradient boosting classifiers based on full Set A of features are shown. They complement the results shown in Table 4 and Figure 2. As we can see, the majority of the points is concentrated at the edges of the plots, corresponding to a situation with at most two non-vanishing class probabilities for given trajectories. The points located near the vertices depict the trajectories with one dominant class. There is much less of a burden in case of the gradient boosting classifier—the probability of assigning a trajectory to a finally claimed class is much higher and there are almost no trajectories with non-zero probabilities for all classes. This is clearly linked to the construction of both these classifiers. In random forest, each base classifier independently returns a predicted class and the final output is the most frequent class returned. Thus, the spread of the predictions can be high. In gradient boosting, the trees are constructed sequentially: each new one is supposed to correct the predictions of the ensemble and its results have a higher weight in the final aggregation. Thus, the final trees are having the greatest impact on the outcome and we expect GB to produce output with one dominant probability in most of the cases.

**Figure 6:**
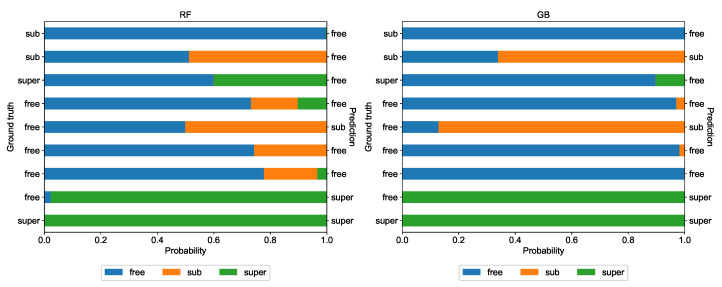
The class probabilities for exemplary trajectories from the testing set, based on the classifiers trained on the base data set and constructed with Set A of features.

In Figure 6, predicted class probabilities for sample trajectories are shown, for random forest (left graph) and gradient boosting (right graph). Indeed, the gradient boosting classifier was more decisive, producing more univocal results, even if they were incorrect (please see the first trajectory from the top and the second trajectory form the bottom).

**Figure 7:**
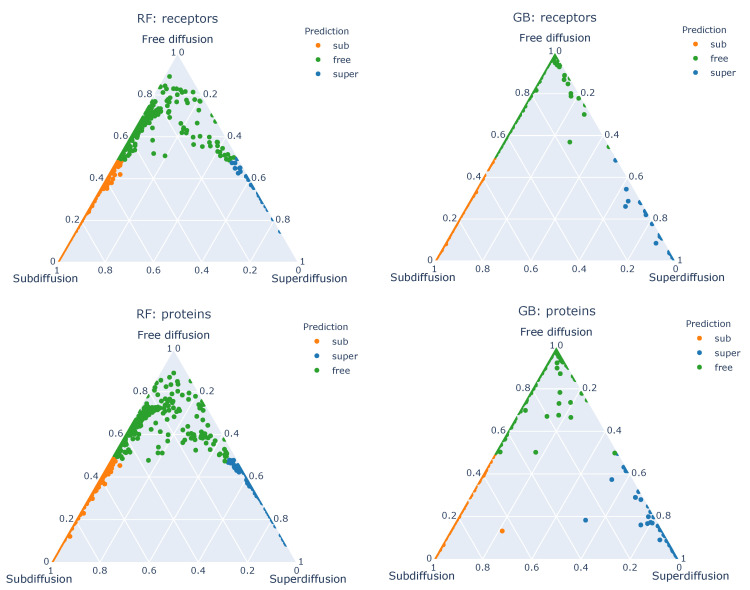
Ternary plots of the class probabilities assigned to empirical data by the classifiers trained on the base data set with Set A of features.

Finally, we can verify the distribution of the class probabilities for our experimental data (see Section 3.3 and Section 4.4), where the ground truth for the diffusion type is not known. In Figure 7, the corresponding ternary plots for empirical data are presented, for random forest and gradient boosting classifiers (left and right column, respectively) and for both G-protein-coupled receptors and G proteins (top and bottom row, respectively). These graphs can clearly show us the trajectories for which the classifiers’ decisions were the most vague—all points near the center of the triangle correspond to trajectories with significant probabilities of all of three diffusion types. Moreover, we can see that in case of random forest, the trajectories classified as superdiffusion had also a significant probability of being a normal diffusion, whereas the gradient boosting classifier undoubtedly returned high probability of them belonging to superdiffusion.

**Figure 8:**
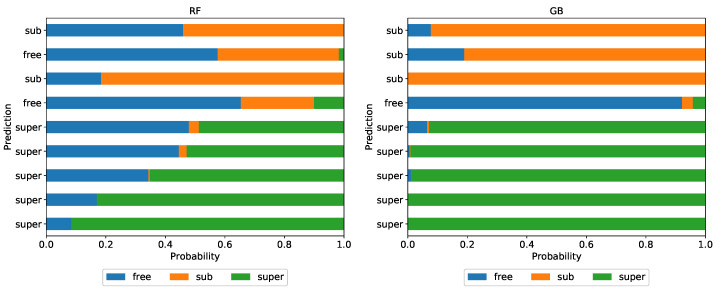
The class probabilities for exemplary trajectories from the empirical data set, based on the classifiers trained on the base data set and constructed with Set A of features.

In Figure 8, the predicted class probabilities for several interesting trajectories are shown, for both random forest (left graph) and gradient boosting (right graph). Again, the gradient boosting algorithm is more firm, but in cases of misclassification, it also claims the incorrect diffusion type with less doubt. Such an analysis of the classifiers decisions is a great starting point for further research—the output classifiers build on different data sets and with different sets of features can be examined in detail to find the exact source of a given prediction. That can also lead us to a reasonable model for the anomaly detection in the trajectories.

## 5. Conclusions

In this paper, we presented a new set of features (referred to as Set A, see Table 1) for the two types of machine learning classifiers, random forest and gradient boosting, that on the synthetic data set gives good results, better than the set used previously in [43]. We have analysed the performance of our classifier trained and tested on the multiple versions of the synthetic data set, allowing us to assess its usefulness, flexibility and robustness. Moreover, we compared the proposed set with the ones already used in this problem, from [40,41,43]. Our set gives the best results in terms of the most common metrics.

Although the results on the synthetic data set are promising, we acknowledge the challenge with the application of the classifiers to real data. As discussed in [41], the classifiers trained on particular models for given diffusion modes do not generalise well. In Section 4.4, we show that even the classifiers with good accuracy return not clear result when used with the data of potentially different characteristics. To some extent, it can be improved by including more models in the training data set.

Thus, we would like to underline the importance of the features’ selection for a given problem—even for the same task (e.g., diffusion mode classification), both models chosen for the training data generation and features chosen for their characterisation have a great influence on the performance of classifiers. Moreover, the assumptions made in constructions of the classifiers, such as hyperparameters’ values or simply the choice of classifier type, are also highly important.

## Figures and Tables

**Table 1:** Features used for classification purposes in each of analysed sets.

Set A	Set B	Set C
	(from [41])	(from [43])
Anomalous exponent α	Anomalous exponent α	Anomalous exponent α
Diffusion coefficient *D*	Diffusion coefficient *D*	Diffusion coefficient *D*
MSD ratio	MSD ratio	—
Efficiency	Efficiency	—
Straightness	Straightness	—
VAC (for lag 1)	—	—
Maximal excursion	—	—
*p*-variation-based statistics	—	—
—	Asymmetry	—
—	Fractal dimension	—
—	Gaussianity	—
—	Kurtosis	—
—	Trappedness	—
—	—	Standardised maximum distance
—	—	Exponent of power function
		fitted to *p*-variation (for p=1,2,…,5)

**Table 2:** Characteristics of the simulated trajectories used to train the classifiers. For the base training set, the following values were used: c=0.1, $\sigma =1\;\mu m\;s^{-1/2}$ and Δt=1s.

Diffusion Class	Model	Parameter Ranges	Number of Trajectories
Normal diffusion	FBM	H∈[0.5−c,0.5+c]	20,000
	DBM	$v=\left(v_{1},v_{2}\right),\;v_{1},v_{2}\in \left[0,c\right]$	10,000
	OU	$\theta =0,\;\lambda =\left(\lambda_{1},\lambda_{2}\right),\;\lambda_{1},\lambda_{2}\in \left[0,c\right]$	10,000
Subdiffusion	FBM	H∈[0.1,0.5−c)	20,000
	OU	$\theta =0,\;\lambda =\left(\lambda_{1},\lambda_{2}\right),\;\lambda_{1},\lambda_{2}\in \left(c,1\right]$	20,000
Superdiffusion	FBM	H∈(0.5+c,0.9]	20,000
	DBM	$v=\left(v_{1},v_{2}\right),\;v_{1},v_{2}\in \left(c,1\right]$	20,000

**Table 3:** Hyperparameters of the optimal classifiers built on base data set with Set A of features. The full set of features is labelled as “with *D*”. The “no *D*“ columns stand for the reduced set of features after the removal of the diffusion coefficient *D*. N/A (i.e., “Not Applicable”) indicates hyperparameters specific for random forest.

	Random Forest		Gradient Boosting	
Hyperpareameters	With *D*	No *D*	With *D*	No *D*
bootstrap	True	True	N/A	N/A
criterion	gini	entropy	N/A	N/A
max_depth	80	10	50	10
max_features	sqrt	sqrt	sqrt	$log_{2}$
min_samples_leaf	4	2	4	2
min_samples_split	2	10	10	2
n_estimators	800	600	900	100

**Table 4:** Accuracy of the best classifiers trained on the base data set (see Table 2) with Set A of features. The “with *D*” and “no *D*” columns refer to the full and reduced (after removal of *D*) sets of features, respectively. The results are rounded to three decimal digits.

	Random Forest		Gradient Boosting	
Data Set	With *D*	No *D*	With *D*	No *D*
Training	0.979	0.962	1.0	0.993
Test	0.957	0.955	0.956	0.955

**Table 5:** Permutation feature importances of the classifiers built on base data set with Set A of features. The “with *D*” and “no *D*” columns refer to the full and reduced (after removal of *D*) sets of features, respectively. The rows are sorted according to the decreasing importances for random forest with *D*. The most and least important features are indicated with bold or underlining, respectively.

	Random Forest		Gradient Boosting	
Feature	With *D*	No *D*	With *D*	No *D*
χ – VAC for δ=1, n=1	**0.1428**	**0.0812**	**0.1612**	**0.2292**
Anomalous exponent α	0.0212	0.0436	0.0244	0.0204
MSD ratio	0.0128	0.0194	0.0080	0.0168
Efficiency	0.0118	0.0074	0.0030	0.0046
Straightness	0.0110	0.0062	0.0064	0.0048
*p*-variation statistic *P*	0.0104	0.0090	0.0024	0.0060
Maximal excursion	0.0080	0.0046	0.0068	0.0094
D	0.0074	–	0.0056	–

**Table 6:** Precision, recall and F1 scores of the classifiers trained on base synthetic data with Set A of features. For each classifier, the testing set consists of 12,000 trajectories per diffusion mode—that is, 36,000 in total. All classifiers were built on base data set with Set A of features.

Method	Variant	Measure	Normal Diffusion	Subdiffusion	Superdiffusion	Total/Average
RF		Precision	0.929	0.973	0.970	0.957
	with *D*	Recall	0.944	0.966	0.962	0.957
		F1	0.936	0.969	0.966	0.957
		Precision	0.922	0.971	0.971	0.955
	no *D*	Recall	0.943	0.963	0.958	0.955
		F1	0.933	0.967	0.964	0.955
GB		Precision	0.928	0.972	0.970	0.956
	with *D*	Recall	0.942	0.966	0.961	0.956
		F1	0.935	0.969	0.965	0.956
		Precision	0.925	0.970	0.969	0.955
	no *D*	Recall	0.940	0.964	0.960	0.955
		F1	0.932	0.967	0.965	0.955

**Table 7:** Accuracy of the classifiers built on the base data set using different sets of features, measured using tenfold cross-validation method. All results are rounded to three decimal digits.

	Random Forest		Gradient Boosting	
Data Set	With *D*	No *D*	With *D*	No *D*
Set A	0.957	0.955	0.956	0.953
Set B	0.946	0.928	0.945	0.928
Set C	0.948	0.946	0.948	0.944

**Table 8:** Hyperparameters of the optimal classifiers built on base data set used for the calculation of tenfold cross-validation accuracy in Table 7. The “with *D*” and “no *D*” columns refer to the full and reduced (after removal of *D*) sets of features, respectively. N/A stands for “Not Applicable” (the first two parameters are random forest specific). The definitions of the feature sets are given in Table 1.

Features	Model	Variant	Bootstrap	Criterion	max_depth	max_features	min_samples_leaf	min_samples_split	n_estimators
Set A	RF	with *D*	True	gini	80	sqrt	4	2	800
		no *D*	True	entropy	10	sqrt	2	10	600
	GB	with *D*	N/A	N/A	50	sqrt	4	10	900
		no *D*	N/A	N/A	10	log2	2	2	100
Set B	RF	with *D*	True	entropy	None	None	2	5	1000
		no *D*	True	entropy	None	log2	1	10	600
	GB	with *D*	N/A	N/A	110	log2	2	10	400
		no *D*	N/A	N/A	10	log2	4	5	100
Set C	RF	with *D*	True	entropy	60	log2	4	2	900
		no *D*	True	entropy	10	sqrt	2	10	600
	GB	with *D*	N/A	N/A	10	log2	2	2	100
		no *D*	N/A	N/A	10	log2	2	2	100

**Table 9:** Detailed performance comparison of random forest classifiers based on three sets of features, built on the base data set. Metrics are calculated on the test data. All results are rounded to three decimal digits. For each classifier, the test set consists of 12,000 trajectories per diffusion mode—that is, 36,000 in total.

Set of Features	Measure	Normal Diffusion	Subdiffusion	Superdiffusion	Total/Average
	Precision	0.929	0.973	0.970	0.957
Set A	Recall	0.944	0.966	0.962	0.957
	F1	0.936	0.969	0.966	0.957
	Precision	0.910	0.970	0.963	0.948
Set B	Recall	0.934	0.957	0.950	0.947
	F1	0.922	0.964	0.956	0.947
	Precision	0.912	0.969	0.966	0.949
Set C	Recall	0.935	0.958	0.951	0.948
	F1	0.923	0.963	0.959	0.948

**Table 10:** Performance of the classifiers trained on data with random Gaussian noise. Accuracies (for test data only) are rounded to three decimal digits.

	Random Forest		Gradient Boosting	
Features	With *D*	No *D*	With *D*	No *D*
Set A	0.950	0.937	0.949	0.937
Set B	0.941	0.918	0.941	0.918
Set C	0.944	0.932	0.943	0.930

**Table 11:** Performance of the classifiers trained on data with σ=0.38. Accuracies (for test data only) are rounded to three decimal digits.

	Random Forest		Gradient Boosting	
Features	With *D*	No *D*	With *D*	No *D*
Set A	0.961	0.959	0.960	0.958
Set B	0.949	0.927	0.948	0.928
Set C	0.953	0.951	0.952	0.949

**Table 12:** Classification results for real trajectories. The base data set (σ=1, no noise; see Section 4.1) with the full sets features (labelled as “with *D*” in the previous sections) was used for training. The numbers may not add up precisely to 100% due to rounding.

		Random Forest		Gradient Boosting	
Features	Classified Mode	Receptor	G Protein	Receptor	G Protein
	Free diffusion	22%	26%	12%	17%
Set A	Subdiffusion	76%	64%	84%	70%
	Superdiffusion	1%	9%	2%	12%
	Free diffusion	2%	7%	0%	0%
Set B	Subdiffusion	97%	90%	99%	97%
	Superdiffusion	0%	1%	0%	2%
	Free diffusion	40%	45%	41%	40%
Set C	Subdiffusion	59%	52%	57%	54%
	Superdiffusion	0%	1%	1%	5%

**Table 13:** Classification results for real trajectories. The noisy data set (σ=1, see Section 4.3) with the full sets of features (labelled as “with *D*” in the previous sections) was used for training. The numbers may not add up precisely to 100% due to rounding.

		Random Forest		Gradient Boosting	
Features	Classified Mode	Receptor	G Protein	Receptor	G Protein
	Free diffusion	28%	31%	27%	28%
Set A	Subdiffusion	70%	61%	70%	61%
	Superdiffusion	1%	6%	2%	9%
	Free diffusion	3%	11%	2%	9%
Set B	Subdiffusion	96%	86%	96%	87%
	Superdiffusion	0%	1%	0%	3%
	Free diffusion	45%	48%	41%	41%
Set C	Subdiffusion	54%	49%	58%	53%
	Superdiffusion	0%	1%	0%	5%

**Table 14:** Classification results for real trajectories. The data set with σ=0.38 (no noise) and with the full sets of features was used for training. The numbers may not add up precisely to 100% due to rounding.

		Random Forest		Gradient Boosting	
Features	Classified Mode	Receptor	G Protein	Receptor	G Protein
	Free diffusion	42%	40%	36%	35%
Set A	Subdiffusion	56%	54%	61%	58%
	Superdiffusion	1%	5%	1%	5%
	Free diffusion	51%	38%	44%	24%
Set B	Subdiffusion	44%	50%	37%	44%
	Superdiffusion	3%	10%	17%	30%
	Free diffusion	54%	51%	54%	51%
Set C	Subdiffusion	45%	47%	45%	46%
	Superdiffusion	0%	1%	0%	1%

**Table 15:** Classification results for real trajectories. The classifiers were trained with the reduced Set A (labelled as “no *D*”). The numbers may not add up precisely to 100% due to rounding.

		Random Forest		Gradient Boosting	
Classifier	Classified Mode	Receptor	G Protein	Receptor	G Protein
	Free diffusion	33%	35%	32%	30%
Base classifier	Subdiffusion	65%	59%	65%	59%
	Superdiffusion	0%	5%	2%	9%
	Free diffusion	72%	58%	77%	60%
Trained with noise	Subdiffusion	25%	34%	18%	29%
	Superdiffusion	1%	6%	3%	10%
	Free diffusion	34%	34%	28%	30%
Trained with σ=0.38	Subdiffusion	63%	58%	69%	59%
	Superdiffusion	1%	7%	2%	10%

**Table 16:** Accuracies on test sets for the classifiers built with the features’ sets with 10% or 50% of MSD curve length used for calculation of the MSD-based features. All results are rounded to three decimal digits.

	Random Forest		Gradient Boosting	
Features	10%	50%	10%	50%
Set A	0.957	0.956	0.956	0.955
Set B	0.947	0.942	0.947	0.942
Set C	0.948	0.947	0.947	0.946

**Table 17:** Accuracies on test set of the optimal classifiers built on data sets with different values of parameter *c* and Set A of features. All results are rounded to three decimal digits.

	Random Forest		Gradient Boosting	
Data Set	With *D*	No *D*	With *D*	No *D*
c=0	0.920	0.919	0.920	0.919
c=0.001	0.924	0.924	0.923	0.923
c=0.01	0.929	0.929	0.928	0.926
c=0.1 (base)	0.957	0.955	0.956	0.955

**Table 18:** Precision, recall and F1 scores for classifiers trained on data with different values of the cutoff *c*. Set A of features was used. All results are rounded to three decimal digits. For each data set, the support of the testing set is 12,000 trajectories per diffusion mode, giving 36,000 in total.

*c* Value	Measure	Normal Diffusion	Subdiffusion	Superdiffusion	Total/Average
	Precision	0.835	0.972	0.974	0.927
c=0	Recall	0.950	0.910	0.900	0.920
	F1	0.889	0.940	0.936	0.921
	Precision	0.842	0.975	0.972	0.930
c=0.001	Recall	0.952	0.915	0.906	0.924
	F1	0.894	0.944	0.938	0.925
	Precision	0.850	0.976	0.976	0.934
c=0.01	Recall	0.955	0.918	0.913	0.929
	F1	0.900	0.946	0.943	0.930
	Precision	0.929	0.973	0.970	0.957
c=0.1	Recall	0.944	0.966	0.962	0.957
	F1	0.936	0.969	0.966	0.957

**Table 19:** Performance of the best classifiers trained on the data set with varying diffusion coefficient *D* and Set A of features. Accuracies are rounded to three decimal digits.

	Random Forest		Gradient Boosting	
Data Set	With *D*	No *D*	With *D*	No *D*
Training	0.971	0.921	0.979	0.966
Test	0.919	0.912	0.920	0.909

## Supplementary Materials

Python codes for every stage of the classification procedure, together with a short documentation, are publicly available at Zenodo (https://doi.org/10.5281/zenodo.4317214).

## Abbreviations

The following abbreviations are used in this manuscript:

DBM	directed Brownian motion
DL	deep learning
FBM	fractional Brownian
GB	gradient boosting
ML	machine learning
MSD	mean square displacement
OU	Ornstein–Uhlenbeck process
RF	random forest
SPT	single-particle tracking
TAMSD	time-averaged mean square displacement
